# Endovascular treatment of giant hepatic hemangiomas: a systematic review and meta-analysis

**DOI:** 10.1590/1677-5449.202500242

**Published:** 2025-12-12

**Authors:** Sofia Malosso Tronconi, João Felipe Federici de Almeida, Ana Terezinha Guillaumon

**Affiliations:** 1 Universidade Estadual de Campinas – Unicamp, Faculdade de Ciências Médicas – FCM, Hospital de Clínicas, Campinas, SP, Brasil.

**Keywords:** therapeutic embolization, endovascular treatment, hepatic hemangioma, systematic review, meta-analysis

## Abstract

Giant hepatic hemangiomas are benign vascular lesions that may cause abdominal pain, compression of adjacent structures, bleeding, and thrombotic complications. Management of symptomatic cases is challenging, especially for larger lesions. This systematic review and meta-analysis evaluated the efficacy and safety of endovascular treatment through transarterial embolization, focusing on the embolization agents employed, reductions in tumor diameter, and associated complications. The results showed significant reductions in mean hemangioma diameter, with low rates of severe complications. This treatment emerges as a promising minimally invasive option for symptomatic or unresectable cases.

## INTRODUCTION

A hepatic hemangioma is a congenital vascular malformation predominantly characterized by vascular ectasia and caused by abnormal angiogenesis. Structurally, it is surrounded by a pseudocapsule. While these are benign tumors, they can expand excessively, compressing adjacent structures or causing other complications, such as obstruction of the bile ducts, compression of the hepatic vein, and provoking Budd-Chiari syndrome, or thrombocytopenic syndrome (Kasabach-Merritt syndrome).^1^

The majority of hepatic hemangiomas are asymptomatic. When symptomatic, their clinical presentation may be related to distension of Glisson’s capsule or to compression of neighboring structures. Diagnosis is frequently incidental during routine examinations and can be made with ultrasonography or abdominal tomography with contrast. Tomography with contrast will show peripheral and nodular filling during the arterial phase, with filling of the entire tumor during the venous phase, and nodule emptying during the late phase.^2^

Historically, considering the last 20 to 30 years, giant hemangiomas were defined as those with maximum diameter greater than or equal to 5 cm. However, more recent publications now suggests a cutoff point of 10 cm, since 5 cm tumors often have no clinical significance. In this study, we reviewed the literature published since 1978, adopting the classic criterion of 5 cm to maintain consistency with previous studies.^1,3-5^

Hepatic hemangioma is more prevalent in women, with growth related to presence of estrogen, whether endogenous or exogenous, which could be the reason for the higher incidence in women.^6^ However, the European Association for the Study of the Liver guidelines do not consider use of oral contraceptives to be contraindicated.^7^ Treatment is indicated in cases of lesions that cause symptoms (such as abdominal pain, nausea, or bloating), progressive growth, or increased risk of bleeding.^1,7-9^

In relation to surgical treatment, both open surgery and laparoscopy may be associated with complications such as bleeding, biliary fistula, infection, and postoperative ileus.^10-12^ Moreover, some large lesions may be considered unresectable, in some cases demanding liver transplantation for treatment.^13-19^ Robotic surgery for resection of hemangiomas has also been described, but experience is limited to date.^10^

Transarterial embolization, whether in isolation or as part of a combined treatment approach, has been growing in popularity as a less invasive option for treatment of several types of hepatic tumors.^20,21^ While intra-arterial embolizations have been described for this purpose since 1978, use of this method has been increasing over recent years.^22^ In the case of hepatic hemangiomas, embolization is performed via selective catheterization of the arteries feeding the tumor, using a range of different embolization agents.^23-25^ The objective of this systematic review is to assess the safety and efficacy of this treatment for management of giant hepatic hemangiomas.

## OBJECTIVES

To conduct a systematic review to assess treatment exclusively by embolization of giant hepatic hemangiomas in adults, excluding emergency scenarios. Additionally, the efficacy and safety of this type of treatment are also assessed.

## METHODS

### Identification of articles

**Figure 1:**
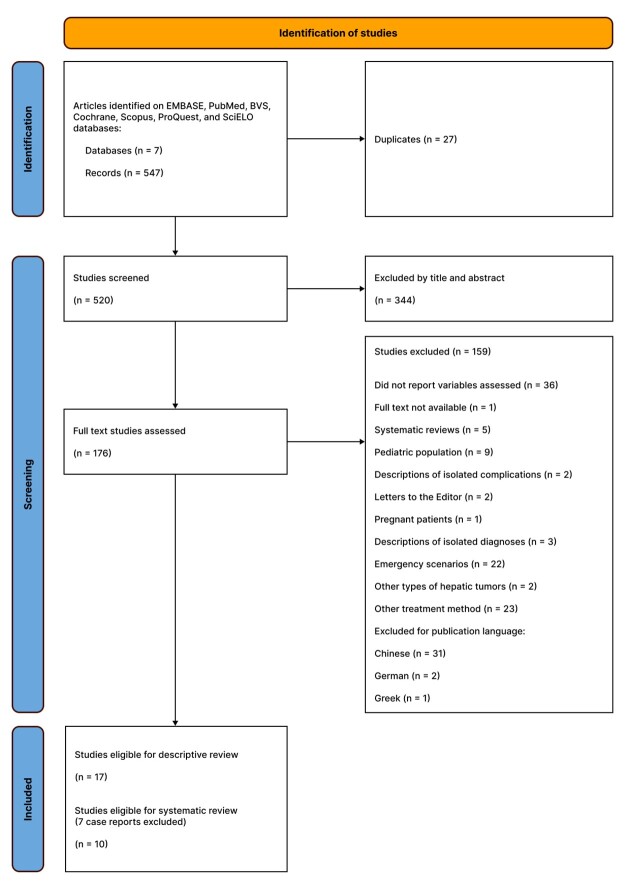
PRISMA 2020 flow diagram. PRISMA = Preferred Reporting Items for Systematic reviews and Meta-Analyses; BVS = Biblioteca Virtual em Saúde.

Articles were identified in databases with the aid of a clinical librarian and artificial intelligence (ChatGPT-4o, openAI), used to identify synonyms and all keywords pertinent to the study. Searches were run on the EMBASE, PubMed, Biblioteca Virtual em Saúde, Cochrane, Scopus, ProQuest, and SciELO databases, with no restrictions on publication date or language. The articles identified were imported into the Mendeley references platform, where they were checked for duplicates. The files were then imported to the Rayyan platform, which is a reference manager with screening tools, for additional duplicate exclusions and subsequent detailed review of the articles. After removal of duplicates, articles were assessed by title and abstract by two reviewers on the Rayyan platform, with a third reviewer intervening in cases of disagreement. After the initial exclusion round, the remaining articles were assessed in full by the lead author of this study. The systematic review was conducted in accordance with the Preferred Reporting Items for Systematic Reviews and Meta-Analyses (PRISMA) 2020, including application of the checklist and construction of a flowchart (Figure 1).^26^ A detailed protocol was designed for this review and is available on request.

### Study selection method

After assessment of the titles and abstracts, the full texts of the remaining studies were analyzed to determine their eligibility for the systematic review.

Studies meeting the following criteria were included:

- Exclusive endovascular transarterial treatment for giant hepatic hemangiomas.
- Patients over 18 years old treated electively.
- No restriction on size of study population.

Description of aspects of the hemangioma before and after the procedure, including:

- Embolization agent used.
- Measurements of the tumor diameter before and after embolization.
- Time elapsed before control examination.

Studies were excluded if they met one or more of the following criteria:

- Treatments conducted in emergency scenarios.
- Treatments combined with surgery, radiotherapy, or percutaneous methods.
- Pregnant patients or children under 18 years of age.
- Publications in languages other than Portuguese, English, or Spanish.
- Literature reviews, Letters to the Editor, or other articles with no original data.
- Publications with no full text available for assessment or purchase.
- Studies that did not report the assessment variables stipulated above.

Studies that did not describe:

- The embolization agent used.
- Tumor diameter before and after the procedure.

### Data collection

Data were collected using a simple table. The data collected were: author, year of publication, country of publication, number of patients, number of hemangiomas, sex, mean age, mean maximum diameter of tumor at diagnosis, mean maximum diameter of tumor after embolization, time elapsed from procedure to control examination, anatomic location of hemangioma, single or multiple lesions, presence of symptoms, type of embolization agent used, number of embolizations needed, and complications.

### Statistical analysis

Initially, a descriptive analysis was conducted of all the studies included in the review, including case reports. Means were calculated using weighted means, with weighted standard deviation (WSD). Although the case reports were not included in the meta-analysis because of insufficient patient numbers, they were still described qualitatively to contribute additional information relevant to the clinical context. When conducting the meta-analysis, it was decided to exclude studies with fewer than eight patients. This decision was based on the need to minimize the disproportional impact of small studies on the quantitative analysis, since they often have very low variance or no standard deviation (SD), which could distort the overall results. Only studies with eight or more patients were included in the meta-analysis, to ensure a more robust statistical analysis and increase the representativeness of the results. F statistic analysis was performed using Python, version 3.11, with support from the NumPy, Pandas, Matplotlib, and SciPy libraries.

## RESULTS

Initially, a total of 547 records were identified for screening. After exclusion of 25 duplicates using Mendeley, 522 studies remained. Another two duplicates were then removed using Rayyan, totaling 520 unique studies. Next, the titles and abstracts were reviewed, excluding 344 studies that did not meet the inclusion criteria. At this point, 176 full text articles were selected for complete assessment. One hundred and fifty-nine (159) of these were excluded after reading the full texts, for the reasons shown in Figure 1, resulting in a total of 17 studies included in the qualitative synthesis, as illustrated in the flow diagram (Figure 1).

### Descriptive analysis

The characteristics of a total of 1,435 patients were assessed in the 17 studies selected. Of these, 507 (35.3%) were men and 928 (64.7%) were women. Single tumors were found in 919 (64.0%) patients, while multiple tumors were found in 372 (26.0%). For 144 (10.0%) patients, no information was provided on whether lesions were single or multiple. Mean patient age was 44.15 (WSD = 2.37 years).

Mean hemangioma diameter before embolization was 9.51 (WSD = 0.83 cm), and mean diameter after embolization had reduced to 3.93 cm (WSD = 1.31 cm). The mean number of procedures per patient was 1.48 (WSD = 0.24), ranging from 1 to 4 embolization sessions. The mean time before follow-up to assess diameter after embolization was 15.06 months (WSD = 20.61 months). The majority of studies used three-phase abdominal tomography for follow-up.

All patients were treated via a transarterial route, using a range of different types of embolization agents. The distribution of agents used was as follows: Lipiodol (iodized oil) + Bleomycin: 285 patients; Lipiodol + Pingyangmycin: 961 patients; Lipiodol + Bleomycin + Gelfoam® particles: 103 patients; Lipiodol + Pingyangmycin + Gelfoam® particles: 53 patients; Microspheres: one patient; Polyvinyl alcohol (PVA) particles: 23 patients; Gelfoam® particles: one patient; Gelfoam® particles + PVA particles + coils: eight patients.

With regard to complications, post-embolization syndrome (PES) was reported in almost all studies, manifesting with abdominal pains, nausea, vomiting, shivering, and fever. Transient increase in hepatic enzymes were also observed, resolving in 2-7 days. Other complications included liver abscess in four patients, transitory jaundice in three, ischemic cholecystitis in three, and late hepatectomy due to liver abscess (5 years after embolization) in one case. No deaths were reported.

### Efficacy analysis

#### Results of the meta-analysis

After data collection for the systematic review, a meta-analysis was conducted to assess the efficacy of transarterial embolization for treatment of giant hepatic hemangiomas. This analysis considered the differences in mean diameter before and after embolization, with their respective standard deviations, taking into account the weight of each study as proportional to its population. This statistical analysis assessed heterogeneity in order to define the best statistical method and generate a forest plot and a funnel plot to visualize and evaluate the results. Studies with fewer than eight patients were excluded from the quantitative analysis (seven case reports), leaving 10 studies included in the meta-analysis, because of the risk of disproportional impact on the combined results and the lack of statistical variance. Case reports are only covered in the descriptive analysis.

#### Assessment of heterogeneity

Heterogeneity among the 10 studies eligible for the meta-analysis was assessed using Cochran’s Q test and the I^2^ statistic, in order to determine the most appropriate statistical model for the meta-analysis. The value of I^2^ was 98.1% (Q = 473.28; degrees of freedom = 9), indicating an elevated level of heterogeneity. Based on these results, a random effects model was chosen, which is more appropriate when studies have substantial differences in characteristics and methods.

In the random effects model, the difference between weighted mean diameters before and after embolization was 5.9 cm. The standard error was 0.031 cm, the 95% confidence interval (95%CI) was 5.84-5.96 cm, with p < 0.0001, indicating statistically significant reduction in hemangioma diameters after embolization. This consistent reduction across studies underscores the efficacy of transarterial embolization as a treatment strategy. The combined mean effect of each study, weighted by their statistical weight, was used to plot the graphs and is also shown in Table 1.

**Table 1:** Data from the 10 studies included in the meta-analysis.

**Author**	**Population**	**Number of tumors**	**Mean diameter before (cm)**	**Standard deviation before**	**Mean diameter after (cm)**	**Standard deviation after**	**Mean difference**	**Standard error of difference**	**CI lower limit**	**CI upper limit**	**P value**	**Weight**	**Weighted mean difference**	**Q value per study**
Zhao et al.^27^	102	109	8.50	3.90	5.90	3.80	2.60	0.53	1.54	3.65	< 0.0001	3.44	8.94	37.74
Yuan et al.^28^	241	241	9.50	3.10	2.90	1.20	6.60	0.21	6.18	7.01	< 0.0001	21.80	143.94	10.31
Shi et al.^29^	53	53	7.10	1.51	4.32	2.18	2.78	0.36	2.06	3.49	< 0.0001	7.53	20.95	73.94
Kirnap et al.^30^	17	19	14.72	12.80	7.63	4.76	7.09	3.31	0.59	13.58	0.0323	0.09	0.64	0.12
Sun et al.^20^	27	27	11.20	5.10	7.60	3.90	3.60	1.23	1.17	6.02	0.0035	0.65	2.35	3.50
Firouzian et al.^31^	20	25	9.70	4.70	8.89	4.32	0.81	1.42	-1.98	3.6	0.5704	0.49	0.39	12.77
Bozkaya et al.^23^	26	32	9.72	0.80	7.63	0.90	2.09	0.23	1.62	2.55	< 0.0001	17.93	37.47	261.97
Zen et al.^32^	98	98	9.70	2.30	3	1.20	6.70	0.26	6.18	7.21	< 0.0001	14.56	97.56	9.03
Srivastava et al.^33^	9	9	9.28	5.13	8.62	2.77	0.66	1.94	-3.14	4.46	0.7341	0.26	0.17	7.30
Li et al.^34^	836	1120	9.60	0.80	3.60	0.50	6	0.03	5.93	6.0	< 0.0001	939.32	5635.95	7.22

CI = confidence interval.

**Figure 2:**
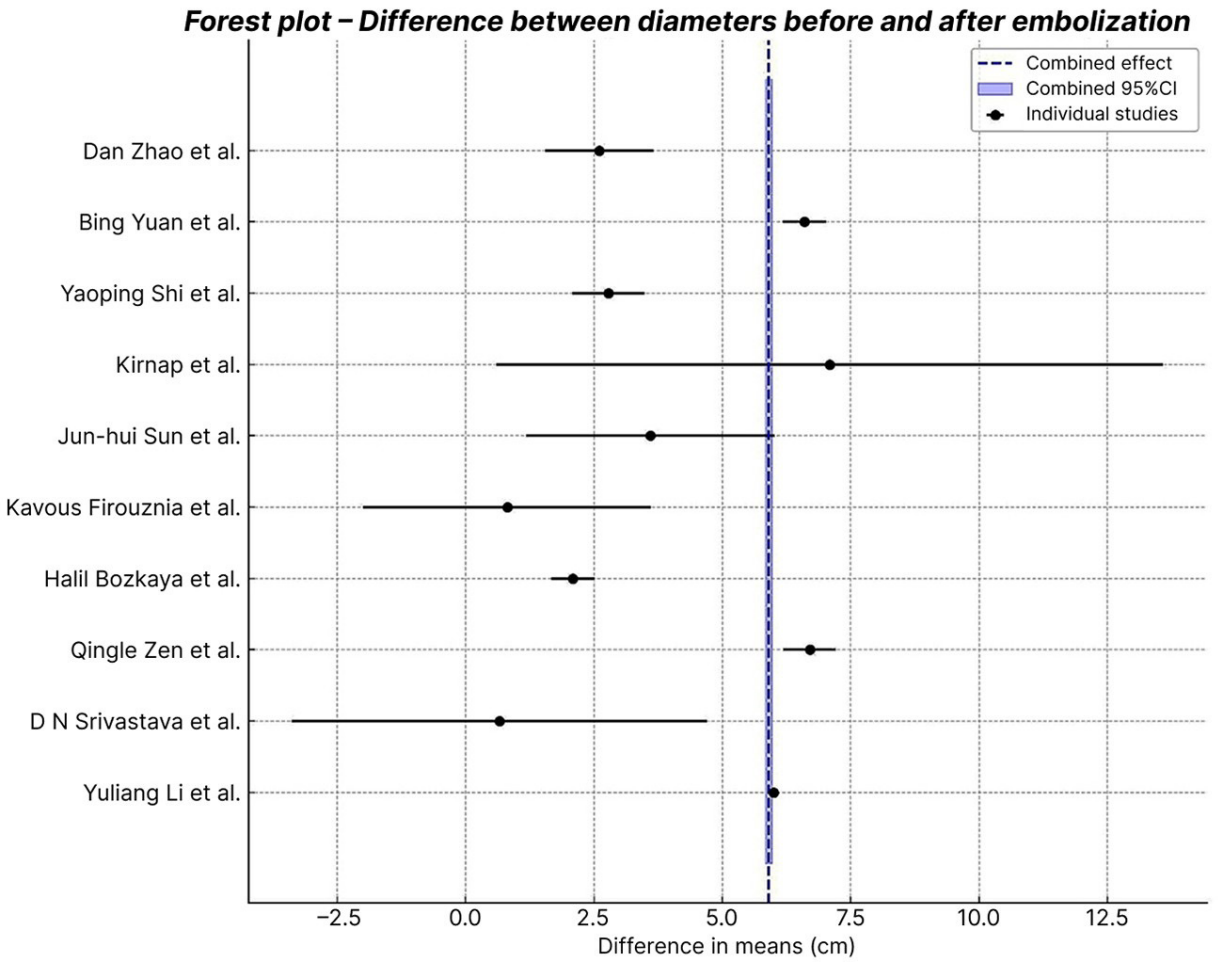
Forest plot.

The results are illustrated graphically in a forest plot (Figure 2). This shows the variability between studies, with some reporting larger reductions in diameter than others. The combined effect shows a statistically significant reduction in diameter, as described above.

**Figure 3:**
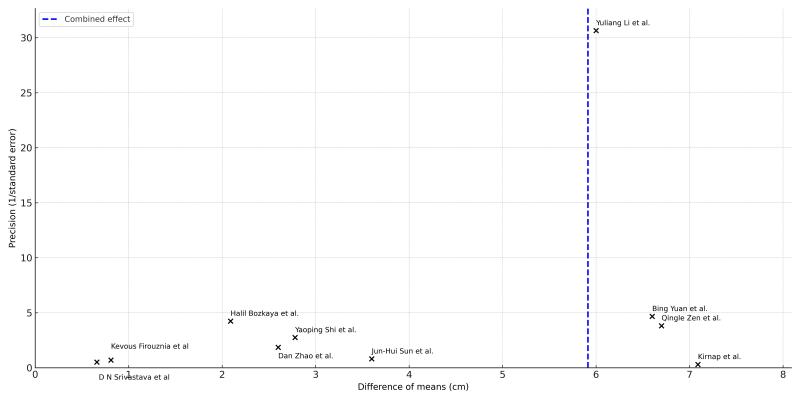
Funnel plot.

The funnel plot (Figure 3) was used to check for publication bias. This graph has an asymmetrical distribution, suggesting possible publication bias. This asymmetry was confirmed with the Egger test, which returned an intercept of -3.98 and p < 0.0001, indicating statistically significant evidence and suggesting the presence of bias. Moreover, the fact that there were few studies with large numbers of patients may have affected the asymmetry of this graph.

Table 2 lists all the variables analyzed in the 17 articles assessed in this study, including the case reports.

**Table 2:** Data from 17 studies, including the case reports.

**Author**	**Country**	**Year of publication**	**Total population +**	**Number of tumors**	**Men**	**Women**	**Mean age (years)**	**Mean diameter at diagnosis (cm)**	**Anatomic location**	**Single**	**Multiple**	**Asymptomatic**	**Symptomatic**	**Mean diameter at follow-up (cm)**	**Time until follow-up CT (months)**	**Embolization agent**	**Number of embolizations needed**
Kurniawan et al.^35^	Indonesia	2024	1	2	0	1	48	9 (SD = 4.24)	Right lobe (1) Left lobe (1)	0	1	0	1	4.50 (SD = 2.12)	4	Bleomycin + Lipiodol	1
Zhao et al.^27^	China	2024	102	109	34	68	47.50	8.50 (SD = 3.90)	ND	95	7	15	87	5.90 (SD = 3.80)	4.50	Lipiodol + Bleomycin + Gelfoam particles	1-3
Zhang et al.^36^	China	2023	1	1	0	1	48	27	Left lobe (1)	1	0	0	1	8.80	12	Lipiodol + Bleomycin + Gelfoam particles	3
Yuan et al.^28^	China	2022	241	ND	52	189	47	9.50 (SD = 3.10)	Right lobe (71) Left lobe (19) Both (151)	42	199	139	102	2.90 (SD = 1.20)	60	Bleomycin + Lipiodol	ND
Shi et al.^29^	China	2020	53	ND	19	34	ND	7.10 (SD = 1.50)	ND	35	18	27	23	4.32 (SD = 2.18)	22	Pingyangmycin + Lipiodol + Gelfoam particles	1-2
Ketchum et al.^37^	Hawaii	2019	1	1	0	1	34	17	Left lobe (1)	1	0	0	1	17	3	Microspheres	1
Kirnap et al.^30^	Turkey	2018	17	19	7	10	46.41	14.72 (SD = 12.80)	Right lobe (7) Left lobe (2) Both (8)	6	11	0	17	7.63 (SD = 4.76)	14.47	Bleomycin + Lipiodol	1-2
Li et al.^34^	China	2015	836	1120	301	535	42.83	9.60 (SD = 0.80)	ND	720	116	0	836	3.60 (SD = 0.50)	4.40	Pingyangmycin + Lipiodol	1-2
Sun et al.^20^	China	2015	27	ND	6	21	47.7	11.20 (SD = 5.10)	Right lobe (13) Left lobe (1) Both (13)	10	17	23	4	7.60 (SD = 3.90)	6	Pingyangmycin + Lipiodol	1
Firouznia et al.^31^	Iran	2014	20	25	4	16	46.8	9.70 (SD = 4.70)	Right lobe (17) Left lobe (8)	ND	ND	5	15	8.89 (SD = 4.32)	6	PVA particles	1
Bozkaya et al.^23^	Turkey	2014	26	32	5	21	49.83	9.72 (SD = 0.80)	Right lobe (24) Left lobe (4) Both (4)	ND	ND	0	26	7.63 (SD = 0.76)	7	Bleomycin + Lipiodol	1-2
Zen et al.^32^	China	2004	98	ND	72	26	41.60	9.70 (SD = 2.30)	Right lobe (61) Left lobe (16) Both (21)	ND	ND	45	53	3 (SD = 1.200)	9	Pingyangmycin + Lipiodol	1
Giavroglou et al.^38^	Greece	2003	1	1	0	1	28	10	Right lobe (1)	1	0	0	1	7	36	PVA particles	1
Giavroglou et al.^38^	Greece	2003	1	1	1	0	58	9	Left lobe (1)	0	1	0	1	8	54	PVA particles	1
Srivastava et al.^33^	India	2001	8	9	5	3	47.75	9.28 (SD = 5.13)	Right lobe (5) Left lobe (1) Both (2)	7	1	0	8	8.62 (SD = 2.77)	9	Gelfoam particles + PVA particles + Coils	1
Althaus et al.^39^	United States	1996	1	2	0	1	29	5.20 (SD = 1.84)	Right lobe (1) Left lobe (1)	0	1	0	1	5.500 (SD = 0.71)	16	PVA particles	1
Panis et al.^40^	France	1993	1	1	1	0	28	17.50	Right lobe (1)	1	0	0	1	12	60	Gelfoam particles	1

CT = computed tomography; ND = not described; PVA = polyvinyl alcohol; SD = standard deviation.

## DISCUSSION

Transarterial embolization as the exclusive treatment for giant hepatic hemangiomas appears to be a valid and effective method for treating this disease. The analysis showed that transarterial embolization is associated with a significant reduction in the diameter of giant hepatic hemangiomas, with a combined effect of 5.9 cm (standard error = 0.031 cm; 95%CI 5.84-5.96 cm, p < 0.0001). This treatment is a promising option and is attractive in comparison to other options such as extensive hepatectomies or liver transplantation.^14,15,17,28^ However, the absence of standardization of embolization agents and the techniques employed highlights the need for further studies to establish optimized protocols and assess the long-term effects of this intervention.^27,32,36,39,40^

There was no mortality associated with treatment of this disease in the studies included in this analysis. Some studies failed to describe the exact technique used for embolization, but the safety of the procedure is linked to the degree of selectivity, with greater safety when superselective catheterization and microcatheters are used to precisely deliver the embolization agent.^28,41^

With regard to the choice of embolization agents, the literature describes a wide range of combinations. The first descriptions used Gelfoam® particles and PVA^33,40^ and use of chemotherapy agents such as Bleomycin and its derivatives has been described more recently.^27,42^ One study reported failure to reduce the size of a 17 cm tumor after embolization with microspheres, which was the only study analyzed that used this type of agent. This case was not included in the meta-analysis, since it was a single case report. Microspheres are not a conventional agent for treatment of hemangioma.^37^

Transarterial embolization of hepatic hemangiomas generally involves a mixture of Lipiodol with Bleomycin or other agents, frequently using the Tessari technique to guarantee homogeneity, since these substances do not mix easily. Proportions and doses can vary depending on tumor size. The most common methods include a combination of 15 to 45 UI of Bleomycin with 7 to 20 ml of Lipiodol, at proportions of 1:1, 1:1.5, or 1:2. The Bleomycin is pre-diluted in saline solution or 5% dextrose solution. Another possible combination is an emulsion of Pingyangmycin, which can also be dissolved in a 5% dextrose solution (2-10 ml) and combined with Lipiodol at proportions ranging from 1:1.5 to 1:3. Additionally, microparticles of substances such as PVA, varying from 100 to 700 µm, can be used to amplify the embolizing effect. Administration is performed slowly via a microcatheter, under fluoroscopic guidance, and is halted when stagnation of arterial flow is observed or when the total volume has been administered.^20,23,27-32,34-36,38,41,43-46^

Pingyangmycin (or Bleomycin A5 hydrochloride) is an antitumor glycoprotein isolated from the several glycoproteins that comprise Bleomycin and is produced by *Streptomyces verticillus var. pingyangensis n.sp.*. Manufactured in China, but unavailable in Brazil and the United States, it is a treatment option at some centers in China and has proven safe and effective over the short term.^20,34^

The largest number of patients treated in any of the articles included in this meta-analysis was described in a multicenter study in China, involving 836 patients with symptomatic giant hepatic hemangiomas who were treated exclusively with transarterial embolization using Pingyangmycin and Lipiodol. This study described a 100% success rate for improvement of symptoms and a significant reduction in mean hemangioma diameter, from 9.6 (SD = 0.8) to 3.6 cm (SD = 0.5). Mean follow-up was 4.4 years (SD = 1.8), with maximum duration of 10 years. During follow-up, two cases of hepatic abscesses were recorded, which were resolved by percutaneous drainage combined with antibiotic therapy, with no other serious complications described.^34^

Long-term monitoring of patients treated with chemoembolization is important, especially in relation to possible late side effects of intra-arterial embolizations. These include vascular endothelial injury, acute or chronic inflammation, and histological fibrosis, associated with use of chemotherapy agents. Bleomycin is widely used in oncology and can cause interstitial pulmonary fibrosis in cumulative high doses. However, the doses administered for embolization remain below the threshold associated with pulmonary effects, even over multiple sessions, (308 UI of intravenous Bleomycin cumulatively).^47,48^

Lipiodol is an iodinated contrast medium indicated for intra-arterial hepatic administration that can be used up to maximum doses of 0.25 mL/kg per session (15-20 mL), although some studies have reported safe use up to 30 mL. Although it is widely used, care is needed with patients with portal hypertension, since it can exacerbate this condition. Precautions must be taken in patients with prior liver failure, because of the increased risk of complications.^28,44^

The heterogeneity of embolization agents and dosages limit direct comparisons between studies, preventing determination of the superiority of any specific approach.^30,31,34^ Prospective studies that consider not only hemangioma size, but also the patients’ clinical symptoms, are essential to establish the best endovascular strategy and assess the long-term safety profiles of embolization agents.

Angiographic assessment of hepatic hemangiomas may be a diagnostic challenge. Some studies describe specific patterns, such as “blooming flowers”, which can help to identify these tumors. However, interpretation of these images demands experience and can impact the choice of treatment strategy.^49^

Postembolization syndrome is a common complication after transarterial embolization of hepatic hemangiomas and is characterized by symptoms such as abdominal pains, fever, and nausea, which usually emerge in the first 24 to 72 hours. While self-limiting, these symptoms require appropriate management with analgesics, antipyretics, and clinical monitoring. Prophylactic wide spectrum antibiotics are also frequently recommended, especially in patients at greater risk of developing hepatic abscesses, such as those with portal hypertension or people who have undergone vascular manipulation with extensive embolization.^36,50^ Studies suggest that the tissue necrosis and local hypoxia induced by embolization may predispose to formation of abscesses.^28,29^ The incidence of PES ranges from 20 to 60% and is influenced by the type of embolization agent, the technique used, and the characteristics of the patient. Use of Bleomycin and Lipiodol is associated with reduced severity of the syndrome, while solid particles, such as PVA, have greater inflammatory potential.^27,44^

This review’s limitations include the inability to assess data from 53 articles published in other languages, the majority in Chinese. Additionally, the retrospective studies included may be susceptible to reporting bias, potentially affecting assessment of outcomes. We decided to limit the review to cases treated electively, excluding ruptured hemangiomas.

## CONCLUSIONS

Based on the results obtained, transarterial embolization proved effective for treatment of giant hepatic hemangiomas, resulting in significant reductions in tumor diameter, with a difference in weighted means of 5.9 cm (standard error = 0.031 cm; 95%CI 5.84-5.96 cm; p < 0.0001). The procedure has a favorable safety profile, with zero reported mortality and a low incidence of serious complications, such as hepatic abscesses. The results indicate that this technique is a viable and less invasive alternative to more aggressive surgical procedures. Moreover, several different embolization agents were described, with combinations involving Lipiodol and Bleomycin or Pingyangmycin standing out for their efficacy for reducing tumor diameter and controlling symptoms. These findings underscore the therapeutic potential of transarterial embolization for management of this condition.

## Data Availability

Todos os dados gerados ou analisados estão incluídos neste artigo e/ou no material suplementar.

## Material Suplementar

Este artigo acompanha material suplementar.

Tabela S1. PRISMA Checklist 2020.

Este material está disponível como parte da versão online do artigo na página https://doi.org/10.1590/1677-5449.202500241
