# Supplementary material for: Endovascular treatment of giant hepatic hemangiomas: a systematic review and meta-analysis
Source: J Vasc Bras. 2025 Dec 12;24:e20250024. doi: 10.1590/1677-5449.202500242 (PMC12704812; doi:10.1590/1677-5449.202500242)
Supplement: Translation

# Translation: Tratamento endovascular de hemangiomas hepáticos gigantes: uma revisão sistemática com metanálise

**DOI:** 10.1590/1677-5449.202500241

**Authors:** Sofia Malosso Tronconi, João Felipe Federici de Almeida, Ana Terezinha Guillaumon

**Affiliations:** 1 Universidade Estadual de Campinas – Unicamp, Faculdade de Ciências Médicas – FCM, Hospital de Clínicas, Campinas, SP, Brasil.

**Keywords:** embolização terapêutica, tratamento endovascular, hemangioma hepático, revisão sistemática, metanálise

## Abstract

Hemangiomas hepáticos gigantes são lesões vasculares benignas que podem causar dor abdominal, compressão de estruturas adjacentes, hemorragia e complicações trombóticas. O manejo dos casos sintomáticos é desafiador, especialmente em lesões maiores. Este estudo é uma revisão sistemática com metanálise que avalia a eficácia e a segurança do tratamento endovascular por meio de embolização transarterial, destacando os agentes embolizantes utilizados, a redução do diâmetro tumoral e as complicações associadas. Foi constatada redução significativa no diâmetro médio dos hemangiomas, com baixa taxa de complicações graves. Esse tratamento configura-se como uma opção minimamente invasiva promissora em casos sintomáticos ou irressecáveis.

## INTRODUÇÃO

O hemangioma hepático é uma malformação vascular congênita caracterizada predominantemente por ectasia vascular, em detrimento de angiogênese. Estruturalmente, encontra-se envolvido por uma pseudocápsula. Apesar de ser um tumor benigno, pode expandir-se excessivamente e comprimir estruturas adjacentes ou causar outras complicações, como obstrução das vias biliares, compressão da veia hepática, levando à síndrome de Budd-Chiari, ou síndrome trombocitopênica (síndrome de Kasabach-Merritt)^1^.

A maioria dos hemangiomas hepáticos é assintomática. Quando sintomático, sua apresentação clínica pode estar relacionada à distensão da cápsula de Glisson ou à compressão de estruturas vizinhas. O diagnóstico, frequentemente incidental em exames de rotina, pode ser realizado por meio de ultrassonografia ou tomografia de abdome com contraste. A tomografia com contraste evidencia enchimento periférico e nodular na fase arterial, com enchimento de toda a lesão na fase venosa e esvaziamento da nodulação na fase tardia^2^.

Historicamente, considerando os últimos 20 a 30 anos, o hemangioma gigante era definido como aquele com diâmetro máximo maior ou igual a 5 cm. Contudo, publicações recentes sugerem um ponto de corte de 10 cm, já que lesões de 5 cm frequentemente carecem de significância clínica. Neste estudo, revisamos a literatura desde 1978, adotando o critério clássico de 5 cm para manter consistência com estudos prévios^1,3-5^.

O hemangioma hepático é mais prevalente em mulheres, apresentando crescimento relacionado à presença de estrógeno, seja endógeno ou exógeno, o que pode explicar a maior incidência nesse grupo^6^. Entretanto, o uso de contraceptivos orais não é contraindicado, conforme as diretrizes da European Association for the Study of the Liver^7^. O tratamento é indicado em casos de lesões sintomáticas (como dor abdominal, náuseas ou plenitude gástrica), crescimento progressivo ou risco aumentado de sangramento^1,7-9^.

Em relação ao tratamento cirúrgico, tanto a cirurgia aberta quanto a laparoscopia podem estar associadas a complicações, como sangramento, fístula biliar, infecção e íleo paralítico pós-operatório^10-12^. Além disso, lesões volumosas podem ser consideradas irressecáveis, demandando, em alguns casos, transplante hepático como alternativa terapêutica^13-19^. A cirurgia robótica para ressecção de hemangiomas também já foi relatada, porém com experiências ainda mais limitadas^10^.

A embolização transarterial, tanto de forma isolada quanto em terapias combinadas, tem ganhado popularidade como uma alternativa menos invasiva para o tratamento de diversos tumores hepáticos^20,21^. Embora embolizações intra-arteriais com esse propósito tenham sido descritas desde 1978, a utilização desse método tem sido ampliada nos últimos anos^22^. No caso dos hemangiomas hepáticos, a embolização é realizada por meio da cateterização seletiva das artérias nutridoras da lesão, utilizando uma variedade de agentes embolizantes^23-25^. Esta revisão sistemática tem como objetivo avaliar a segurança e a eficácia desse tratamento no manejo de hemangiomas hepáticos gigantes.

## OBJETIVOS

Realizar uma revisão sistemática para avaliar o tratamento exclusivamente por embolização de hemangioma hepático gigante em adultos, excluindo contextos de urgência. Além disso, avaliar a eficácia e a segurança desse tipo de tratamento.

## MÉTODOS

### Identificação dos artigos

A identificação dos artigos nas bases de dados foi realizada com o auxílio de uma bibliotecária clínica e da inteligência artificial (ChatGPT-4o, openAI), utilizada para identificar sinônimos e todas as palavras-chave pertinentes ao estudo. As buscas foram conduzidas nas bases de dados EMBASE, PubMed, Biblioteca Virtual em Saúde, Cochrane, Scopus, ProQuest e SciELO, sem restrições quanto à data de publicação ou ao idioma. Os artigos encontrados foram importados para a plataforma de referências Mendeley, onde foi realizada a revisão de duplicatas. Após esse processo, os arquivos foram adicionados à plataforma Rayyan, um gerenciador de referências e ferramenta de triagem, para exclusão adicional de duplicatas e posterior revisão pormenorizada dos artigos. Após a remoção das duplicatas, os artigos foram avaliados por título e resumo por dois revisores na plataforma Rayyan, com intervenção de um terceiro revisor nos casos de discordância. Após a primeira etapa de exclusão, os artigos restantes foram avaliados na íntegra pela pesquisadora principal deste trabalho. Esta revisão sistemática foi conduzida de acordo com as diretrizes Preferred Reporting Items for Systematic Reviews and Meta-Analyses (PRISMA) 2020, incluindo a *checklist* e o diagrama de fluxo (Figura 1)^26^. Um protocolo detalhado desta revisão foi elaborado e está disponível mediante solicitação.

**Figura 1:** Fluxograma do método PRISMA 2020. PRISMA = Preferred Reporting Items for Systematic reviews and Meta-Analyses; BVS = Biblioteca Virtual em Saúde.

### Método de seleção dos estudos

Após a avaliação de títulos e resumos, os estudos restantes foram analisados na íntegra para determinar sua elegibilidade na presente revisão sistemática.

Foram incluídos estudos que atendiam aos seguintes critérios:

- Tratamento endovascular transarterial exclusivo de hemangiomas hepáticos gigantes.
- Pacientes maiores de 18 anos, tratados em contexto eletivo.
- Sem restrição quanto ao tamanho da população estudada.

Descrição de aspectos do hemangioma antes e após o procedimento, incluindo:

- Agente embolizante utilizado.
- Medidas do diâmetro da lesão pré- e pós-embolização.
- Intervalo até a realização do exame de controle.

Foram excluídos estudos que apresentavam uma ou mais das seguintes características:

- Tratamentos realizados em contexto de urgência.
- Tratamentos combinados com cirurgia, radioterapia ou métodos percutâneos.
- Pacientes gestantes ou menores de 18 anos.
- Publicações em idiomas diferentes de português, inglês ou espanhol.
- Estudos do tipo revisão de literatura, carta ao editor ou outros artigos sem dados originais.
- Publicações não disponíveis para avaliação completa ou compra.
- Estudos que não reportavam as variáveis estipuladas previamente para avaliação.

Estudos que não descreviam:

- O agente embolizante utilizado.
- Medidas do diâmetro da lesão antes e após o procedimento.

### Coleta de dados

A coleta de dados foi realizada utilizando uma tabela simples. Os dados coletados foram: autor, ano de publicação, país de publicação, número de pacientes, número de hemangiomas, sexo, idade média, média do diâmetro máximo da lesão ao diagnóstico, média do diâmetro máximo da lesão após embolização, tempo para exame de controle após o procedimento, localização anatômica do hemangioma, solitário ou múltiplo, presença de sintomas, tipo de agente embolizante utilizado, número de embolizações necessárias e complicações.

### Análise estatística

Inicialmente, foi realizada uma análise descritiva de todos os estudos incluídos na revisão, incluindo os relatos de caso. As médias foram calculadas utilizando média ponderada, com descrição do desvio-padrão ponderado (DPP). Os relatos de caso, embora não tenham sido incluídos na metanálise devido ao número insuficiente de pacientes, foram descritos qualitativamente para agregar informações relevantes ao contexto clínico. Para a realização da metanálise, optou-se por excluir estudos com menos de oito pacientes. Essa decisão foi baseada na necessidade de minimizar o impacto desproporcional de estudos pequenos na análise quantitativa, já que esses frequentemente apresentam variância muito baixa ou ausência de desvio-padrão (DP), o que poderia distorcer os resultados globais. Apenas estudos com oito ou mais pacientes foram considerados na metanálise, a fim de garantir maior robustez estatística e representatividade dos resultados. A análise estatística foi realizada utilizando o software Python, versão 3.11, com suporte das bibliotecas NumPy, Pandas, Matplotlib e SciPy.

## RESULTADOS

Inicialmente, foram identificados 547 registros para triagem. Após a exclusão de 25 duplicatas pelo *software* Mendeley, restaram 522 estudos. Posteriormente, mais duas duplicatas foram removidas com o uso do Rayyan, totalizando 520 estudos únicos. Em seguida, realizou-se a revisão de títulos e resumos, na qual 344 estudos foram excluídos por não atenderem aos critérios de inclusão. Assim, 176 artigos foram selecionados para avaliação completa. Destes, 159 foram excluídos após leitura integral do texto, conforme justificativas apresentadas na Figura 1, resultando em 17 estudos finais incluídos na síntese qualitativa, como descrito no fluxograma abaixo (Figura 1).

### Análise descritiva

Dos 17 estudos selecionados, foram avaliadas características de um total de 1.435 pacientes. Destes, 507 (35,3%) eram homens e 928 (64,7%) mulheres. Lesões únicas foram observadas em 919 (64,0%) pacientes, enquanto lesões múltiplas foram observadas em 372 (26,0%). Em 144 (10,0%) pacientes, não foi discriminado se as lesões eram únicas ou múltiplas. A média de idade foi de 44,15 (DPP = 2,37 anos).

O diâmetro médio dos hemangiomas antes da embolização foi de 9,51 (DPP = 0,83 cm), e após a embolização esse diâmetro médio foi reduzido para 3,93 cm (DPP = 1,31 cm). O número médio de procedimentos realizados por paciente foi de 1,48 (DPP = 0,24), variando de 1 a 4 sessões de embolização. O tempo médio para avaliação do diâmetro após embolização foi de 15,06 meses (DPP = 20,61 meses). Na maioria dos estudos, o controle foi realizado com tomografia de abdome trifásica.

Em todos os pacientes, o tratamento realizado foi via transarterial, com utilização de diversos tipos de agentes embolizantes. A distribuição do uso dos agentes foi a seguinte: Lipiodol (óleo iodado) + Bleomicina: 285 pacientes. Lipiodol + Pingyangmycina: 961 pacientes. Lipiodol + Bleomicina + partículas de Gelfoam®: 103 pacientes. Lipiodol + Pingyangmycina + partículas de Gelfoam®: 53 pacientes. Microesferas: um paciente. Partículas de álcool polivinílico (PVA): 23 pacientes. Partículas de Gelfoam®: um paciente. Partículas de Gelfoam® + partículas de PVA + molas (*coil*): oito pacientes.

Com relação às complicações, a síndrome pós-embolização (SPE) foi relatada em quase todos os estudos, manifestando-se com dor abdominal, náuseas, vômitos, calafrios e febre. Também foi observado aumento transitório de enzimas hepáticas, com resolução entre 2-7 dias. Outras complicações incluíram abscesso hepático em quatro pacientes, icterícia transitória em três, colecistite isquêmica em três e hepatectomia tardia devido a abscesso hepático (após 5 anos da embolização) em um caso. Não houve mortalidade descrita.

### Análise da efetividade

#### Resultados da metanálise

Após a coleta de dados na revisão sistemática, a metanálise foi realizada para avaliar a eficácia da embolização transarterial no tratamento de hemangiomas hepáticos gigantes. Para isso, foi considerada a diferença do diâmetro médio pré- e pós-embolização, com seus respectivos desvios-padrões, levando em conta o peso de cada estudo proporcional à sua população. A análise estatística avaliou também a heterogeneidade, a fim de definir o melhor método estatístico e compor os gráficos de *forest plot* e *funnel plot* para avaliação e visualização dos resultados. Foram excluídos da análise quantitativa estudos com menos de oito pacientes (sete relatos de caso), restando 10 estudos incluídos na metanálise, devido ao risco de impacto desproporcional nos resultados combinados e à falta de variabilidade estatística. Os relatos de caso foram apresentados apenas de forma descritiva na análise.

#### Avaliação da heterogeneidade

A heterogeneidade entre os 10 estudos elegíveis para metanálise foi avaliada por meio do teste Q de Cochran e da estatística I^2^, a fim de determinar o modelo estatístico mais apropriado para a metanálise. O valor de I^2^ foi de 98,1% (Q = 473,28; graus de liberdade = 9), indicando um nível elevado de heterogeneidade. Com base nesses resultados, optou-se pelo uso do modelo de efeitos aleatórios, mais adequado quando os estudos apresentam diferenças substanciais em suas características e métodos.

No modelo de efeitos aleatórios, a diferença de médias ponderadas entre os diâmetros pré- e pós-embolização foi de 5,9 cm. O erro-padrão foi de 0,031 cm, o intervalo de confiança de 95% (IC95%) foi de 5,84-5,96 cm e p < 0,0001, indicando redução estatisticamente significativa do diâmetro dos hemangiomas após a embolização. Essa redução consistente entre os estudos reforça a eficácia da embolização transarterial como estratégia terapêutica. O efeito da média combinada de cada estudo, ponderada pelo seu peso estatístico, foi utilizado para a representação dos gráficos, e também está apresentado na Tabela 1.

**Tabela 1:** Dados dos 10 estudos incluídos na metanálise.

**Autor**	**População**	**Número de lesões**	**Média diâmetro pré (cm)**	**Desvio-Padrão pré**	**Média diâmetro pós (cm)**	**Desvio-Padrão pós**	**Média de diferença**	**Erro-padrão da diferença**	**IC limite inferior**	**IC limite superior**	**Valor de p**	**Peso**	**Média ponderada da diferença**	**Valor de Q por estudo**
Zhao et al.^27^	102	109	8,50	3,90	5,90	3,80	2,60	0,53	1,54	3,65	< 0,0001	3,44	8,94	37,74
Yuan et al.^28^	241	241	9,50	3,10	2,90	1,20	6,60	0,21	6,18	7,01	< 0,0001	21,80	143,94	10,31
Shi et al.^29^	53	53	7,10	1,51	4,32	2,18	2,78	0,36	2,06	3,49	< 0,0001	7,53	20,95	73,94
Kirnap et al.^30^	17	19	14,72	12,80	7,63	4,76	7,09	3,31	0,59	13,58	0,0323	0,09	0,64	0,12
Sun et al.^20^	27	27	11,20	5,10	7,60	3,90	3,60	1,23	1,17	6,02	0,0035	0,65	2,35	3,50
Firouzian et al.^31^	20	25	9,70	4,70	8,89	4,32	0,81	1,42	-1,98	3,60	0,5704	0,49	0,39	12,77
Bozkaya et al.^23^	26	32	9,72	0,80	7,63	0,90	2,09	0,23	1,62	2,55	< 0,0001	17,93	37,47	261,97
Zen et al.^32^	98	98	9,70	2,30	3	1,20	6,70	0,26	6,18	7,21	< 0,0001	14,56	97,56	9,03
Srivastava et al.^33^	9	9	9,28	5,13	8,62	2,77	0,66	1,94	-3,14	4,46	0,7341	0,26	0,17	7,30
Li et al.^34^	836	1120	9,60	0,80	3,60	0,50	6	0,03	5,93	6,06	< 0,0001	939,32	5635,95	7,22

IC = intervalo de confiança.

Os resultados foram apresentados graficamente em um *forest plot* (Figura 2). O gráfico evidencia a variabilidade entre os estudos, com alguns apresentando maiores reduções no diâmetro do que outros. No efeito combinado, observa-se uma redução de diâmetro estatisticamente significativa, conforme descrito previamente.

**Figura 2:** *Forest plot*.

O gráfico de *funnel plot* (Figura 3) foi utilizado para avaliar a presença de viés de publicação. O gráfico apresentou uma distribuição assimétrica, sugerindo a possibilidade de viés de publicação. Essa assimetria foi confirmada pelo teste de Egger, que apresentou um intercepto de -3,98, com p < 0,0001, indicando evidência estatisticamente significativa e sugerindo a presença de viés, além disso, o fato de haver poucos estudos com grande número de pacientes incluídos pode ter influenciado na assimetria do gráfico.

**Figura 3:** *Funnel plot*.

A Tabela 2 detalha todas as variáveis analisadas nos 17 artigos avaliados neste estudo, incluindo os relatos de caso.

**Tabela 2:** Dados dos 17 estudos, incluindo os relatos de caso.

**Autor**	**País**	**Ano de publicação**	**População total**	**Número de lesões**	**Homem**	**Mulher**	**Média idade (anos)**	**Diâmetro médio ao diagnóstico (cm)**	**Localização anatômica**	**Solitário**	**Múltiplo**	**Assintomático**	**Sintomático**	**Diâmetro médio no controle (cm)**	**Tempo para TC de controle (mês)**	**Agente embolizante**	**Número de embolizações necessárias**
Kurniawan et al.^35^	Indonésia	2024	1	2	0	1	48	9 (DP = 4,24)	Lobo direito (1) Lobo esquerdo (1)	0	1	0	1	4,50 (DP = 2,12)	4	Bleomicina + Lipiodol	1
Zhao et al.^27^	China	2024	102	109	34	68	47,50	8,50 (DP = 3,90)	ND	95	7	15	87	5,90 (DP = 3,80)	4,50	Lipiodol + Bleomicina + Partículas de Gelfoam	1-3
Zhang et al.^36^	China	2023	1	1	0	1	48	27	Lobo esquerdo (1)	1	0	0	1	8,80	12	Lipiodol + Bleomicina + Partículas de Gelfoam	3
Yuan et al.^28^	China	2022	241	ND	52	189	47	9,50 (DP = 3,100)	Lobo direito (71) Lobo esquerdo (19) Ambos (151)	42	199	139	102	2,90 (DP = 1,20)	60	Bleomicina + Lipiodol	ND
Shi et al.^29^	China	2020	53	ND	19	34	ND	7,10 (DP = 1,50)	ND	35	18	27	23	4,32 (DP = 2,18)	22	Pingyangmycina + Lipiodol + Partículas de Gelfoam	1-2
Ketchum et al.^37^	Hawai	2019	1	1	0	1	34	17	Lobo esquerdo (1)	1	0	0	1	17	3	Microesferas	1
Kirnap et al.^30^	Turquia	2018	17	19	7	10	46,41	14,72 (DP = 12,80)	Lobo direito (7) Lobo esquerdo (2) Ambos (8)	6	11	0	17	7,63 (DP = 4,76)	14,47	Bleomicina + Lipiodol	1-2
Li et al.^34^	China	2015	836	1120	301	535	42,83	9,60 (DP = 0,80)	ND	720	116	0	836	3,60 (DP = 0,50)	4,4	Pingyangmycina + Lipiodol	1-2
Sun et al.^20^	China	2015	27	ND	6	21	47,70	11,20 (DP = 5,10)	Lobo direito (13) Lobo esquerdo (1) Ambos (13)	10	17	23	4	7,60 (DP = 3,90)	6	Pingyangmycina + Lipiodol	1
Firouznia et al.^31^	Iran	2014	20	25	4	16	46,8	9,70 (DP = 4,70)	Lobo direito (17) Lobo esquerdo (8)	ND	ND	5	15	8,89 (DP = 4,32)	6	Partículas de PVA	1
Bozkaya et al.^23^	Turquia	2014	26	32	5	21	49,83	9,72 (DP = 0,80)	Lobo direito (24) Lobo esquerdo (4) Ambos (4)	ND	ND	0	26	7,63 (DP = 0,76)	7	Bleomicina + Lipiodol	1-2
Zen et al.^32^	China	2004	98	ND	72	26	41,60	9,70 (DP = 2,30)	Lobo direito (61) Lobo esquerdo (16) Ambos (21)	ND	ND	45	53	3 (DP = 1,20)	9	Pingyangmycina + Lipiodol	1
Giavroglou et al.^38^	Grécia	2003	1	1	0	1	28	10	Lobo direito (1)	1	0	0	1	7	36	Partículas de PVA	1
Giavroglou et al.^38^	Grécia	2003	1	1	1	0	58	9	Lobo esquerdo (1)	0	1	0	1	8	54	Partículas de PVA	1
Srivastava et al.^33^	Índia	2001	8	9	5	3	47,75	9,28 (DP = 5,13)	Lobo direito (5) Lobo esquerdo (1) Ambos (2)	7	1	0	8	8,62 (DP = 2,77)	9	Partículas de Gelfoam + Partículas de PVA + Mola	1
Althaus et al.^39^	EUA	1996	1	2	0	1	29	5,20 (DP = 1,84)	Lobo direito (1) Lobo esquerdo (1)	0	1	0	1	5,50 (DP = 0,71)	16	Partículas de PVA	1
Panis et al.^40^	França	1993	1	1	1	0	28	17,50	Lobo direito (1)	1	0	0	1	12	60	Partículas de Gelfoam	1

DP = desvio-padrão; ND = não descrito; PVA = álcool polivinílico; TC = tomografia computadorizada.

## DISCUSSÃO

A embolização transarterial de hemangioma hepático gigante, como terapia isolada, parece ser um método válido e efetivo para o tratamento dessa doença. A análise demonstrou que a embolização transarterial está associada a uma redução significativa no diâmetro dos hemangiomas hepáticos gigantes, com um efeito combinado de 5,9 cm (erro-padrão = 0,031 cm; IC95% 5,84-5,96 cm, p < 0,0001). O tratamento é uma alternativa promissora e torna-se atrativo quando comparado a outras opções, como hepatectomias extensas ou transplante hepático^14,15,17,28^. No entanto, a ausência de padronização dos agentes embolizantes e das técnicas utilizadas destaca a necessidade de estudos adicionais, a fim de estabelecer protocolos otimizados e avaliar os efeitos a longo prazo dessa intervenção^27,32,36,39,40^.

Não houve mortalidade associada ao tratamento dessa doença nos estudos incluídos na presente análise. Alguns estudos falham em descrever a técnica exata da embolização, mas a segurança do procedimento é associada ao grau de seletividade, sendo mais elevada quando realizada por meio de cateterização superseletiva com uso de microcateteres para a entrega precisa do agente embolizante^28,41^.

Em relação à escolha dos agentes embolizantes, a literatura descreve uma ampla variedade de combinações. As primeiras descrições de tratamento utilizaram partículas de Gelfoam® e PVA^33,40^ e, mais recentemente, o uso de agentes quimioterápicos, como a Bleomicina e seus derivados^27,42^. Um estudo descreveu falha na redução do tamanho de um hemangioma de 17 cm após embolização com microesferas, sendo o único estudo analisado a empregar esse tipo de agente. Esse caso não foi incluído na metanálise, já que se tratava de um relato de caso. As microesferas não são agentes convencionais para o tratamento do hemangioma^37^.

A embolização transarterial de hemangiomas hepáticos geralmente envolve a mistura de Lipiodol com Bleomicina ou outros agentes, frequentemente utilizando a técnica de Tessari para garantir a homogeneidade, visto que as substâncias não se misturam facilmente. As proporções e doses podem variar conforme o tamanho do tumor. Os métodos mais comuns incluem a combinação de 15 a 45 UI de Bleomicina com 7 a 20 ml de Lipiodol, em proporções de 1:1, 1:1,5 ou 1:2. Para isso, a Bleomicina é preparada previamente em solução salina ou glicose 5%. Outra combinação possível é a emulsão de Pingyangmycin, que pode ser dissolvida em solução de glicose a 5% (2-10 ml) e combinada ao Lipiodol na proporção de 1:1,5 a 1:3. Adicionalmente, micropartículas como PVA, variando de 100 a 700 µm, podem ser utilizadas para potencializar o efeito embolizante. A administração é feita lentamente por microcateter, sob controle fluoroscópico, sendo interrompida quando se observa estagnação do fluxo arterial ou após o volume total administrado^20,23,27-32,34-36,38,41,43-46^.

A Pingyangmycina, ou hidrocloridrato de Bleomicina A5, é uma glicoproteína antitumoral isolada das várias glicoproteínas que compõem a Bleomicina, sendo produzida pelo *Streptomyces verticillus var. pingyangensis n. sp*. Fabricada na China e não comercializada no Brasil ou nos EUA, é a opção terapêutica em alguns centros chineses, mostrando-se eficaz e segura a curto prazo^20,34^.

O maior número de pacientes tratados entre os artigos incluídos nesta metanálise foi relatado em um estudo multicêntrico realizado na China, envolvendo 836 pacientes com hemangiomas hepáticos gigantes sintomáticos, tratados exclusivamente com embolização transarterial utilizando Pingyangmycina e Lipiodol. Esse estudo descreveu taxa de sucesso de 100% na melhora dos sintomas e redução significativa do diâmetro médio do hemangioma, de 9,6 (DP = 0,8) para 3,6 cm (DP = 0,5). O seguimento médio foi de 4,4 anos (DP = 1,8), com duração máxima de 10 anos. Durante o acompanhamento, dois casos de abscessos hepáticos foram registrados, sendo resolvidos com drenagem percutânea associada à antibioticoterapia, sem outras complicações mais graves descritas^34^.

A monitorização a longo prazo dos pacientes tratados com quimioembolização é importante, especialmente em relação a possíveis efeitos colaterais tardios das embolizações intra-arteriais. Entre esses, destacam-se o dano endotelial vascular, a inflamação aguda ou crônica e a fibrose histológica, associados ao uso de agentes quimioterápicos. A Bleomicina, amplamente utilizada em oncologia, pode causar fibrose pulmonar intersticial em doses cumulativas elevadas. No entanto, as doses administradas para a embolização, mesmo em múltiplas sessões, permanecem abaixo do limiar associado a efeitos pulmonares (308 UI de Bleomicina cumulativa em aplicação endovenosa)^47,48^.

O Lipiodol é um meio de contraste iodado indicado para administração hepática intra-arterial, podendo ser utilizado em doses máximas de 0,25 mL/kg por sessão (15-20 mL), embora alguns estudos tenham descrito doses seguras de até 30 mL. Apesar de amplamente empregado, requer cuidado em pacientes com hipertensão portal, pois pode exacerbar esse quadro. Precauções devem ser tomadas em pacientes com insuficiência hepática prévia, devido ao risco aumentado de complicações^28,44^.

A heterogeneidade dos agentes embolizantes e das dosagens utilizadas limita a comparação direta entre estudos, dificultando a determinação de superioridade de uma abordagem específica^30,31,34^. Estudos prospectivos, que considerem não apenas o tamanho do hemangioma, mas também os sintomas clínicos dos pacientes, são essenciais para estabelecer a melhor estratégia endovascular e avaliar o perfil de segurança a longo prazo dos agentes utilizados.

A avaliação angiográfica dos hemangiomas hepáticos pode apresentar desafios diagnósticos. Alguns estudos descrevem padrões específicos, como o "aspecto de florescimento" (*blooming flowers*), que podem auxiliar na caracterização dessas lesões. No entanto, a interpretação dessas imagens requer experiência e pode influenciar na escolha da estratégia terapêutica^49^.

A SPE é uma complicação frequente após a embolização transarterial de hemangiomas hepáticos, sendo caracterizada por sintomas como dor abdominal, febre e mal-estar, que usualmente surgem nas primeiras 24 a 72 horas. Embora autolimitados, esses sintomas demandam manejo adequado com analgésicos, antipiréticos e monitoramento clínico. O uso profilático de antibióticos de amplo espectro é frequentemente recomendado, especialmente em pacientes com maior risco de evolução para abscessos hepáticos, como aqueles com hipertensão portal ou submetidos a manipulação vascular com embolização extensa^36,50^. Estudos sugerem que a necrose tecidual e a hipóxia local induzidas pela embolização podem predispor à formação de abscessos^28,29^. A incidência de SPE varia entre 20 e 60%, sendo influenciada pelo tipo de agente embolizante, pela técnica utilizada e pelas características do paciente. O uso de Bleomicina e Lipiodol está associado a uma menor severidade da síndrome, enquanto partículas sólidas, como o PVA, apresentam maior potencial inflamatório^27,44^.

Entre as limitações desta revisão, destaca-se a impossibilidade de avaliar dados de 53 artigos publicados em outras línguas, sendo o chinês a mais representativa entre aquelas não analisadas. Além disso, os estudos retrospectivos incluídos podem introduzir viés de relato, potencialmente impactando a avaliação dos desfechos. Optamos por restringir a revisão apenas a casos tratados de forma eletiva, excluindo situações de hemangiomas rotos.

## CONCLUSÃO

Com base nos resultados obtidos, a embolização transarterial mostrou-se eficaz no tratamento de hemangiomas hepáticos gigantes, promovendo uma redução significativa do diâmetro das lesões, com uma diferença de médias ponderadas de 5,9 cm (erro-padrão = 0,031 cm; IC95% 5,84-5,96 cm; p < 0,0001). O procedimento apresentou um perfil de segurança favorável, sem mortalidade relatada e baixa incidência de complicações graves, como abscessos hepáticos. Os resultados indicam que a técnica constitui uma alternativa viável e menos invasiva em comparação a procedimentos cirúrgicos mais agressivos. Além disso, o uso de diferentes agentes embolizantes foi descrito, destacando-se combinações envolvendo Lipiodol e Bleomicina ou Pingyangmycina, que demonstraram efetividade em reduzir o diâmetro tumoral e controlar sintomas. Esses achados reforçam o papel terapêutico da embolização transarterial no manejo dessa condição.

## Data Availability

All data generated or analyzed in this study are included in this article and its supplementary material.

## Supplementary Material

Supplementary material accompanies this paper.

Table S1 PRISMA Checklist 2020.

This material is available as part of the online article from https://doi.org/10.1590/1677-5449.202500242
